# Vitamin D Supplementation: Shedding Light on the Role of the Sunshine Vitamin in the Prevention and Management of Type 2 Diabetes and Its Complications

**DOI:** 10.3390/nu16213651

**Published:** 2024-10-26

**Authors:** Dimitra Vasdeki, Georgios Tsamos, Evangelos Dimakakos, Vasileios Patriarcheas, Theocharis Koufakis, Kalliopi Kotsa, Armand Cholewka, Agata Stanek

**Affiliations:** 1 Division of Endocrinology and Metabolism and Diabetes Centre, First Department of Internal Medicine, Medical School, Aristotle University of Thessaloniki, AHEPA University Hospital, Stilponos Kyriakides 1 St., 54636 Thessaloniki, Greece; demivs14@gmail.com (D.V.); kkalli@auth.gr (K.K.); 2 Second Propedeutic Department of Internal Medicine, Hippokration General Hospital, Aristotle University of Thessaloniki, Konstantinoupoleos 49 St., 54942 Thessaloniki, Greece; tsamgeor@gmail.com (G.T.); thkoyfak@auth.gr (T.K.); 3 Oncology Unit, Third Department of Internal Medicine, Sotiria General Hospital for Chest Diseases, National and Kapodistrian University of Athens, 152 Mesogeion Ave., 11527 Athens, Greece; edimakakos@gmail.com; 4 First Propaedeutic Department of Internal Medicine, Aristotle University of Thessaloniki, AHEPA University Hospital, Stilponos Kyriakides 1 Str., 54636 Thessaloniki, Greece; vpatriar@gmail.com; 5 Faculty of Science and Technology, University of Silesia, Bankowa 14 Street, 40-007 Katowice, Poland; armand.cholewka@us.edu.pl; 6 Department of Internal Medicine and Metabolic Diseases, Faculty of Health Sciences in Katowice, Medical University of Silesia, Poniatowskiego 15 St., 40-055 Katowice, Poland; 7 Upper-Silesian Medical Centre of the Medical University of Silesia in Katowice, Ziołowa 45-46 St., 40-635 Katowice, Poland

**Keywords:** vitamin D, supplements, type 2 diabetes mellitus, glycaemic control

## Abstract

As the incidence of type 2 diabetes mellitus (T2DM) continues to increase globally, researchers are keen to investigate various interventions to mitigate its impact. Among these, vitamin D supplementation has attracted significant attention due to its influence on insulin secretion from the pancreas and insulin receptors in body cells. A substantial body of evidence indicates that vitamin D supplementation can reduce low-grade inflammation, a critical factor in developing insulin resistance. In addition, vitamin D aids in sustaining low resting concentrations of reactive oxygen species and free radicals, normalizes Ca^2+^ signaling, diminishes the expression of cytokines that are pro-inflammatory, and enhances the production of cytokines that are anti-inflammatory. This review discusses the effects of vitamin D on the glycemic control of individuals with T2DM and evaluates the impact of vitamin D supplementation on glycemic markers in this population. The investigation employs a comprehensive analysis of the existing literature with a special focus on recent studies published in the past decade. Based on the findings in the literature, it can be concluded that vitamin D supplementation alongside anti-diabetic medications may enhance glycemic control and potentially reduce the risk of diabetic complications. The evidence supports the notion that vitamin D supplementation can be a valuable addition to pharmacological agents for the management of T2DM, potentially enhancing glycemic control and overall health outcomes in affected individuals.

## 1. Introduction

Type 2 diabetes mellitus (T2DM) is recognized as a global epidemic, with the International Diabetes Federation Global Map reporting that nearly 425 million people worldwide are affected by the condition. This number is projected to increase to approximately 700 million by 2045 [1,2,3]. Over 90% of cases of diabetes are T2DM, which places a significant financial burden on international healthcare systems [4,5]. This condition is marked by a diminished sensitivity to insulin and malfunctioning β-cells in the pancreas that secrete insulin [6]. Diabetes has a substantial impact on an individual’s quality of life and is one of the primary causes of morbidness, premature death, and serious consequences such as sightlessness, coronary artery disease, amputation, and kidney failure [7,8]. Although advances in T2DM remedies have been made in recent decades, ongoing research and new approaches for prevention and management remain essential due to the increasing prevalence of the disease.

Recently, vitamin D (VD) has attracted significant attention due to its pleiotropic effects beyond skeletal health, particularly its associations with cardiovascular and metabolic diseases [9,10,11,12,13,14]. Deficient VD levels appear to increase the risk of developing diabetes mellitus [15]. This association can be partly explained by the presence of VD receptors in more than 30 biological tissues [16]. The enzyme 1α-hydroxylase, which activates VD, is expressed in pancreatic β-cells [17]. Additionally, VD response elements are found in insulin genes, where they enhance the transcription of insulin receptor genes [18,19]. Specific polymorphisms in the VD receptor gene have been linked to metabolic syndrome [20]. VD also regulates nuclear peroxisome proliferator-activated receptors (PPARs), which are crucial for insulin sensitivity [21]. PPARs help reduce the expression of pro-inflammatory cytokines, such as interleukin (IL)-1, IL-6, and tumor necrosis factor (TNF)-α, which are involved in insulin resistance [22]. Furthermore, animal studies have shown that VD is essential for normal insulin secretion [23,24]. 

VD has been hypothesized to possess anti-diabetic properties through several mechanisms, including the regulation of insulin secretion and sensitivity, anti-inflammatory properties, and down-regulation of high parathyroid hormone levels, which can influence insulin secretion [25,26,27,28]. Although some research supports the role of VD in glucose homeostasis, the findings have been inconsistent. Cross-sectional studies have linked insufficient VD levels with the development of type 2 diabetes, its complications, obesity, and metabolic syndrome [29,30,31,32]. A link between low blood VD levels and the occurrence of T2DM or “metabolic syndrome” was discovered by a meta-analysis of data collected through observational studies [33]. However, a separate meta-analysis of four prospective cohort studies found no significant association between VD intake and the risk of developing type 2 diabetes [34]. Despite these mixed results, the potential anti-diabetic effects of VD and the underlying mechanisms warrant further investigation.

In this literature review, we aimed to investigate the protective effects of vitamin D on pancreatic β-cells, elucidate its underlying molecular mechanisms, and highlight the benefits of vitamin D on glycemic control as well as its role in reducing diabetic complications. Our goal is to establish a foundation for the potential development of VD as a therapeutic agent to manage T2DM and its associated complications.

## 2. Unraveling the Molecular Mysteries: How Vitamin D Influences Diabetes Pathophysiology

Primarily recognized for its role in calcium metabolism, VD undergoes conversion to 25(OH)D in the liver and is further metabolized in the kidneys to form 1,25-dihydroxycholecalciferol or 1,25(OH)2D, the active metabolite responsible for facilitating intestinal calcium absorption and bone mineralization. Consequently, severe deficiency in VD is well-known to lead to impaired bone development, manifesting as osteomalacia in adults and rickets in children. However, the role of VD in improving pancreatic β-cell function and insulin sensitivity is less recognized. The mechanism mainly involves promoting insulin receptor expression and activating the peroxisome proliferator-activated receptor-δ, which facilitates glucose uptake in peripheral tissues, similar to the action in the anti-diabetic drug pioglitazone [35]. In addition, VD stimulates insulin secretion by activating VD receptors in pancreatic β-cells [36]. As a result, VD supplementation has been shown to improve fasting glucose and HbA1c levels in patients, as well as to reduce the risk of developing T2DM [37,38].

New and in-depth understandings of the molecular mechanisms via which VD affects important cellular processes and functions have been made possible by recent investigations. VD has been shown to play a beneficial role in conditions where these processes are compromised, including impaired autophagy, dysfunction of mitochondria, chronic and low-grade inflammation, oxidative stress that produces reactive species and radicals, DNA damage, epigenetic changes, and abnormalities in Ca^2+^ signaling. The review by Berridge et al. excellently explores these roles of VD, particularly in the context of aging [39]. A recent meta-analysis spanning studies from 1980 to 2019 has offered significant knowledge of the biochemical pathways through which vitamin D deficiency contributes to the onset and progression of insulin resistance [40]. This comprehensive analysis confirmed and verified that sufficient vitamin D levels significantly mitigate insulin resistance-related cellular pathology. It accomplishes this by sustaining minimal concentrations of reactive species and radicals, facilitating normal Ca^2+^ signaling, reducing the generation of pro-inflammatory cytokines, and enhancing the production of anti-inflammatory cytokines. In addition, VD plays a crucial role in preventing DNA hypermethylation and subsequent inactivation of essential genes, along with mitigating additional epigenetic modifications in β-cells and insulin-responsive peripheral tissues.

Furthermore, the active form of VD, 1,25(OH)_2_ cholecalciferol, plays a crucial role in insulin secretion by β-cells by improving ions of calcium flooding into the intracellular space, a critical process for insulin exocytosis in response to elevated blood glucose levels [41,42]. Calcium homeostasis within these cells is regulated by proteins such as calbindin D28k, which modulate intracellular calcium signals and protect β-cells from apoptosis and necrosis by inhibiting free radical formation [43]. The concentration of biologically active VD is, in turn, influenced by VD-binding protein levels [44]. Additionally, VD regulates insulin action in tissues both directly, by modulating insulin receptor expression, and indirectly, by activating PPARs, nuclear transcription factors involved in fatty acid metabolism in muscle and adipose tissue [45,46,47,48]. Furthermore, there is a significant association between VD deficiency, T2DM, primary hyperparathyroidism, and impaired glucose tolerance [49].

Additionally, VD is closely involved in mitigating oxidative stress, which is characterized by increased quantities of reactive species and radicals in insulin-sensitive organs, including β-cells, in T2DM. Oxidative stress arises from impaired mitochondrial function or inadequate removal of reactive species due to diminished autophagy, leading to cellular damage and inflammation [50]. VD regulates oxidative stress by modulating cellular antioxidants and supporting mitochondrial function, functioning within the VD–klotho–Nrf2 network. It enhances the expression of key genes such as glutamate cysteine ligase and glutathione reductase, which are crucial for glutathione synthesis and reduction, respectively. Furthermore, VD increases glucose-6-phosphate dehydrogenase activity, which produces NADPH, vital for the reduction of oxidized glutathione [40,51]. By increasing superoxide dismutase expression, VD helps reduce nitrogen oxides (NOx) and facilitates the conversion of harmful reactive species into less detrimental forms. Moreover, VD augments the expression of glutathione peroxidase, thereby reducing lipid hydroperoxides and hydrogen peroxide, further contributing to the mitigation of oxidative stress [52,53].

**Figure 1:**
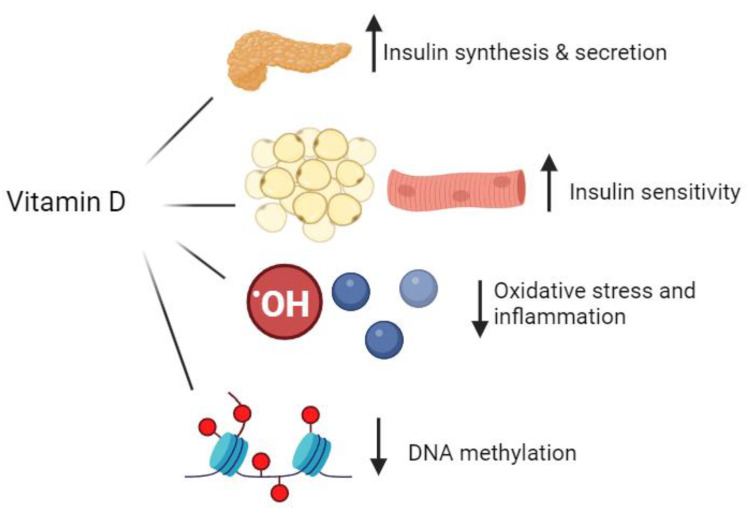
Vitamin D can influence diabetes pathophysiology through several mechanisms: (1) stimulates insulin synthesis and secretion from b-cells, (2) increases insulin sensitivity in peripheral tissues, (3) reduces oxidative stress and inflammation, (4) promotes expression of DNA demethylases, mitigating diabetic epigenetic alterations.

Moreover, compromised mitochondrial function substantially exacerbates chronic low-grade inflammation, a primary contributor to the onset of insulin resistance. VD has the ability to modulate this inflammatory state by reducing the expression of pro-inflammatory cytokines and the enhancement of anti-inflammatory cytokine production. Deficiencies in VD are typically associated with a pro-inflammatory cellular and tissue environment [54,55,56]. Furthermore, VD performs a pivotal role in maintaining the epigenome, where epigenetic changes associated with insulin resistance and T2DM often involve DNA hypermethylation, leading to gene inactivation. VD can counteract these hypermethylation effects by promoting the expression of DNA demethylases, thus mitigating diabetic epigenetic alterations [57,58]. The mechanisms of how VD can influence diabetes pathophysiology are presented in Figure 1. 

Ultimately, VD plays a crucial role in enhancing insulin synthesis and promoting its secretion from pancreatic β-cells [23,59,60]. These cells express VD receptors (VDRs), and a vitamin D response element is found in the promoter region of the human insulin receptor gene [18]. The binding of the active form of VD, 1,25-dihydroxyvitamin D [1,25(OH)_2_D], to VDRs, activates a cascade of genes responsible for β-cell growth, insulin production, and glucose transport [19,61]. In addition to its genomic effects, VD influences insulin secretion through non-genomic mechanisms. It depolarizes the cytoplasmic membranes of β-cells, opening calcium channels, which increases intracellular calcium levels. This calcium influx triggers insulin release through the exocytosis of insulin-containing vesicles. This process highlights the dual genomic and non-genomic roles of VD in insulin regulation. Several studies provide evidence supporting these mechanisms. For example, serum VD levels have been found to correlate positively with insulin sensitivity and β-cell function in healthy populations [62]. Additionally, a study demonstrated that cholecalciferol supplementation (5000 IU daily) for six months significantly improved both peripheral insulin sensitivity and β-cell function, further underscoring VD’s role in glucose homeostasis [63].

## 3. Vitamin D Supplementation: A Game Changer for Glucose Control in Type 2 Diabetes?

As noted previously, VD insufficiency is prevalent in persons with T2DM and can significantly influence the disease’s start by impacting insulin production, fasting levels of glucose, and systemic inflammation [33,64,65]. Several observational studies have shown that people with the lowest serum VD levels face an increased risk of developing diabetes by up to 50% compared to those with the highest levels [66,67]. Various scientific societies, including the Scandinavian Nutrition Societies, the European Society for Clinical and Economic Aspects of Osteoporosis and Osteoarthritis, the North American Institute of Medicine, the German Osteology governing body (DVO), and the D-ACH nutrition societies, have concurred that circulating 25(OH)D levels should not descend below 50 nmol/L, with levels beneath 25–30 nmol/L signifying a deficiency [68,69].

Genetic variables significantly influence the effectiveness of VD supplementation in managing T2DM. A 2023 study identified that non-responders to VD supplementation had specific single nucleotide polymorphisms (SNPs), which may limit their response. This suggests that genetic variability can affect the metabolic pathways involved in VD’s role in glycemic control [70]. Another study demonstrated that serum 25-hydroxyvitamin D levels in diabetic patients after long-term VD_3_ supplementation are associated with CYP27B1 polymorphisms, particularly the rs10877012 G/T allele, which confers a better response to supplementation. These genetic variations offer the potential for personalizing VD intake in individuals with T2DM to ensure a higher efficacy of supplementation [71]. By identifying specific genetic markers, healthcare providers could tailor VD supplementation strategies based on an individual’s genetic predisposition to respond favorably.

In addition to genetic factors, racial differences may influence the effectiveness of VD supplementation. Studies indicate that oral VD absorption is similar in African and Caucasian populations, suggesting that lower VD levels in African individuals may be due to reduced production in the skin rather than differences in absorption [72]. However, other research has shown that conservative oral VD supplementation may be largely ineffective in achieving therapeutic serum levels, particularly in African populations, highlighting the need for higher or alternative forms of supplementation in these groups [73]. Environmental factors, such as air pollution, weather conditions (e.g., cloud cover), ozone levels, and living at high latitudes, also affect VD production, contributing to a higher risk of deficiency [74]. While these factors are well-recognized as contributors to VD deficiency, gaps in the literature persist regarding the variability in response to supplementation.

Future research should focus on developing individualized supplementation strategies, accounting for genetic, racial, and environmental factors. Such strategies could optimize the benefits of VD supplementation, particularly for improving glycemic control and managing diabetes-related complications in diverse populations.

### 3.1. Effect on HbA1c

Several interventional studies have yielded significant findings on the impact of VD supplementation on HbA1c levels. A randomized controlled trial (RCT) conducted by Nikooyeh et al. in 2014 demonstrated a substantial reduction in HbA1c levels following vitamin D administration compared to a placebo [75]. Similarly, a trial with 118 participants, randomized to receive vitamin D alone or combined with calcium or a placebo, observed a significant reduction in HbA1c levels in the VD plus calcium group, while no significant reduction was observed in the group receiving only VD [76]. Furthermore, a pilot RCT conducted by Soric et al. indicated a tendency for a more substantial decrease in HbA1c levels in the VD group relative to the control group, yet this disparity did not achieve statistical significance [77]. Additionally, Nasri et al. observed a notable disparity in HbA1c concentrations between the intervention versus control groups, but this effect was observed exclusively in male patients [78]. Shaseb et al. reported improved fasting blood glucose and glycated hemoglobin levels after a high-dose VD injection in patients with T2DM, while Eftekhari et al. noted increased insulin secretion and improved HbA1c after 12 weeks of VD supplementation [1,79]. Krul-Poel et al. found that only severely VD-deficient individuals saw a reduction in glycated hemoglobin, while Tabesh et al. observed no changes in glycemic markers with isolated VD supplementation but noted improvements when co-supplemented with calcium [76,80].

On the contrary, the findings of several meta-analyses have shown different results. A meta-analysis conducted by Krul-Poel and colleagues revealed no significant change in HbA1c levels following VD supplementation compared to a placebo, a result echoed by Li [81,82]. A study by Morieira-Lucas et al. demonstrated a considerable elevation in 25(OH)D levels, although no alterations in β-cell function, HbA1c, or fasting insulin following 2 years of supplementation with 4000 IU of VD, findings consistent with Wagner et al. [83,84]. However, a recent meta-analysis by Farahmand et al. demonstrated a significant difference in HbA1c levels comparing those receiving the treatment and placebo groups in pooled analyses [85]. Similarly, a meta-analysis conducted by Mirhosseini et al. indicated that a minimum daily dosage of 4000 IU is required to elicit beneficial effects on HbA1c, HOMA-IR, fasting plasma glucose, and to attain adequate circulating levels of 25 (OH) D [82,86].

### 3.2. Effect on Fasting Glucose

Regarding the impact of VD supplementation on fasting glucose, some studies, including those by Shab-Bidar, Nikooyeh, and Jafari, demonstrated a significant reduction [75,87,88]. Pittas et al. found that VD and calcium supplementation could prevent increases in glucose levels and insulin resistance in those with impaired fasting glucose [89]. However, a more recent trial showed significant reductions in fasting blood sugar, glycated hemoglobin, and 2-h blood glucose levels in women with overweightness and prediabetes who were VD deficient [90]. On the contrary, other studies did not report significant changes in fasting glucose concentrations comparing the VD and placebo groups, or the levels remained constant from baseline [91,92,93,94,95,96,97]. A meta-analysis found a significant effect of VD supplementation on fasting glucose only in a specific subgroup of studies, a result that aligns with the findings of Elkassaby et al., who reported a significantly higher decrease in fasting glucose in the VD group compared to the placebo [81,98]. A more recent meta-analysis by Farahmand et al. found a substantial change in fasting glucose levels between the intervention and placebo groups in pooled analyses [85]. However, Lee et al. in 2017 reported that VD supplementation exerted little influence on fasting glucose readings [99]. Therefore, further randomized controlled trials (RCTs) with larger sample sizes and longer durations are needed to gain a clearer understanding of the impact of VD supplementation on fasting glucose in the future.

### 3.3. Effect on Insulin Resistance

Various studies have used the homeostasis model assessment of insulin resistance (HOMA-IR) in individuals with T2DM to evaluate the effect of VD supplementation on glycemic control. More than a decade ago, an RCT conducted by Witham et al. showed that insulin resistance did not improve with VD supplementation, a finding later supported by Jorde et al. and El Hajj, who also reported no significant change [100,101,102]. However, subsequent studies reported a substantial decrease or decline in HOMA-IR after VD administration [1,103,104,105]. For instance, Tabesh et al. observed that calcium and VD co-supplementation resulted in reduced serum insulin and HOMA-IR levels [76]. In contrast, research conducted by Kampmann et al., utilizing the hyperinsulinemic–euglycemic clamp method—regarded as the gold standard for assessing insulin resistance—did not demonstrate a beneficial effect of vitamin D on glycemic control in 16 individuals with T2DM [97]. A randomized trial involving 92 people with prediabetes showed improved insulin secretion with VD and calcium supplementation, although it did not affect insulin resistance [106]. Subsequent comprehensive evaluations have indicated enhancements in insulin resistance and fasting plasma glucose levels in individuals with T2DM after VD administration [107,108]. Nevertheless, a meta-analysis conducted in 2018 reported a reduction in HOMA-IR among intervention groups vs. placebo groups [82]. Table 1 below summarizes some of the studies that examine the effect of VD on HOMA-IR.

These discrepancies in findings stem from various methodological limitations, including differences in VD dosage, supplementation duration, and form, as well as individual factors like baseline serum VD levels, individual responsiveness, and genetic variations affecting VD metabolism. Moreover, the efficacy of VD supplementation may fluctuate based on health conditions, such as whether individuals are diabetic or non-diabetic, the type of diabetes (type 1 or type 2), and other related outcome measures, including diabetes prevalence, blood glucose levels, and insulin concentrations [67,70,109,110].

It is worth mentioning that VD has specific effects on certain populations, including the elderly, middle-aged adults, and minors. For instance, an RCT conducted in 2021 demonstrated that VD supplementation in elderly individuals diagnosed with prediabetes led to significant reductions in fasting glucose levels and HbA1c after 12 months [111]. Additionally, another study highlighted that in a high-risk population such as elderly individuals with prediabetes, a weekly VD supplementation regimen was effective in alleviating symptoms of anxiety and depression [112]. Moreover, a retrospective study focusing on children and adolescents with VD deficiency and either type 1 or type 2 diabetes mellitus reported that VD supplementation was associated with statistically significant decreases in body mass index standard deviation score (BMI SDS), alanine aminotransferase (ALT) levels, and a clinically meaningful reduction in HbA1c [113].

Finally, it is important to highlight that an increased intake of vitamin D supplements, particularly without proper medical supervision, poses a significant risk of exogenous hypervitaminosis D, which leads to vitamin D toxicity (VDT) and is often manifested by hypercalcemia. Symptoms of hypercalcemia include nausea, vomiting, weakness, frequent urination, and in severe cases, kidney damage and bone loss [114]. As VD toxicity is becoming more of a concern with the growing availability and use of high-dose supplements, it is crucial to emphasize the safe upper limit for daily VD intake. According to current evidence, the daily upper safe limit for VD intake is 4000 IU for most adults [115]. Exceeding this threshold, especially over prolonged periods, can be harmful and increase the risk of VDT. Regular monitoring of serum VD and calcium levels, particularly for individuals taking high doses, can help mitigate these risks. Dose adjustments based on individual needs and ongoing health status are essential to ensure safe supplementation and avoid toxicity. Bolus doses greater than 300,000 IU have been associated with an increased risk of hypercalcemia and hypercalciuria and should generally be avoided as a standard practice.

**Table 1:** Summary of the intervention studies examining the effect of VD on glycemic parameters.

Study	Population	Duration	Intervention/Control Group	Outcomes on Glycemic Parameters
Shab-Bidar et al. (2011) [87]	100	12 weeks	Doogh with added vitamin D3 (1000 IU/day plus 340 mg Ca/day) as opposed to plain doogh (340 mg Ca/day)	Decreased fasting glucose and insulin levels. No notable alterations in HbA1c levels.
Nikooyeh et al. (2014) [75]	90	12 weeks	1. Vitamin D3-enriched yoghurt (1000 IU daily) 2. Yogurt enriched with vitamin D3 and calcium (1000 IU/500 mg daily) as opposed to plain yogurt	Decreased levels of HbA1c, HOMA-IR, fasting glucose, and insulin.
Heshmat et al. (2012) [93]	42	3 months	Comparing a single dose of vitamin D3 (300.000 IU) against a placebo	No notable alterations in HbA1c levels. Increased HOMA-IR and fasting glucose levels.
Ryu et al. (2013) [96]	158	24 weeks	Vitamin D3 1000 IU daily and calcium 100 mg twice a day against calcium 100 mg administered bi-daily.	No significant differences were observed in HbA1c or HOMA-IR levels.
Nasri et al. (2013) [78]	60	12 weeks	Vitamin D3 50,000 IU per week versus placebo	Decrease in HbA1c levels among male subjects.
Jehle et al. (2014) [92]	55	6 months	Single dosage of vitamin D3 300,000 IU intramuscularly versus placebo	Decreased HOMA-IR. No notable alterations in fasting insulin and glucose levels were observed. The intervention group exhibited a markedly reduced increase in HbA1c levels.
Kampmann et al. (2014) [97]	16	12 weeks	Vitamin D3 11,200 IU per day during 2 weeks, followed by 5600 IU per day for 10 weeks, compared to placebo.	No notable alterations in insulin sensitivity or HbA1c levels were observed.
Tabesh et al. (2014) [76]	118	8 weeks	1. Vitamin D3 50,000 IU per week 2. Approximately 1000 mg per day 3. Vitamin D3 50,000 IU each week plus calcium 1000 mg per day versus placebo	Reduction in HbA1c, HOMA-IR, fasting glucose, and insulin levels observed in the calcium + vitamin D group. No alterations were observed in the vitamin D group.
Al-Zahrani et al. (2014) [94]	183	3 months	Vitamin D3 45,000 IU per week compared to placebo	No differences observed in HbA1c or fasting glucose levels.
Elkassaby et al. (2014) [98]	52	6 months	Vitamin D3 10,000 IU daily for 2 weeks, followed by 6000 IU daily for 6 months, compared to placebo.	No differences were observed in HbA1c, insulin, or HOMA-IR. However, fasting glucose was significantly lower in the VD group compared to the placebo.
Jafari et al. (2015) [88]	59	12 weeks	Yogurt fortified with Vitamin D3 (2000 IU/day) versus plain yogurt	No notable alterations in HbA1c levels. Decreased fasting glucose, insulin, and HOMA-IR observed.
Krul-Poel et al. (2015) [80]	261	6 months	Vitamin D3 50,000 IU per month versus placebo	No significant changes were observed in HbA1c and HOMA-IR levels. A decrease in HbA1c was noted in the subgroup with 25(OH)D levels at or below 30 nmol/L.
Meta-analysis examining the effect of VD on glycemic parameters.				
Pittas et al., 2007 [33]	N/A	N/A	2000 IU of vitamin D3 per day or 700 IU of vitamin D3 daily with a supplementary dose of 500 mg of calcium citrate each day	Insufficient levels of vitamin D and calcium may adversely affect glycemia, while supplementation with both nutrients may enhance glucose metabolism.
Krul-Poel et al., 2017 [81]	1797	4 to 52 weeks	1000 IU/day of vitamin D3, escalating to 45,000 IU/week, or 11,200 IU/day of vitamin D3 for 2 weeks, followed by 5600 IU/day for 10 weeks, or a single dose ranging from 100,000 to 300,000 IU of vitamin D3.	A notable effect on FBG was observed in a subgroup of studies (n = 4); however, no significant effect was found in the change in HbA1c.
Mirhosseini et al., 2018 [86]	3848	8 to 260 weeks	From 420 IU per day to 88,880 IU per week of vitamin D3	Significant decrease in HbA1c, FBG, and HOMA-IR
Hu et al., 2019 [116]	1374	4 to 24 weeks	Administration of up to 50,000 IU of vitamin D3 weekly or a single injection of 300,000 IU of vitamin D3.	There was a notable decrease in HbA1c, HOMA-IR, and insulin levels in the group receiving short-term vitamin D supplementation.

HbA1c: Glycated Haemoglobin, HOMA-IR: Homeostatic Model Assessment for Insulin Resistance, FBG: fasting blood glucose.

## 4. Impact of Vitamin D Supplementation on Lipid Profile

The lipid profile plays a crucial role in T2DM management because it helps assess the risk of cardiovascular diseases (CVDs), which are significantly more prevalent in diabetic patients. Individuals with T2DM often exhibit an atherogenic lipid profile, characterized by elevated triglycerides, low-density lipoprotein cholesterol (LDL-C), and reduced high-density lipoprotein cholesterol (HDL-C) levels. This combination of lipid abnormalities substantially raises the risk of atherosclerosis and other cardiovascular complications compared to individuals without diabetes. Dyslipidemia in T2DM often goes unnoticed but can have severe consequences [117]. It has been found that patients with diabetes are two to four times more likely to die from heart disease than adults without diabetes, making early detection and management of dyslipidemia critical [118].

It has been well-established that deficient VD levels are associated with unfavorable serum lipid profiles, while adequate VD levels correlate with healthier lipid profiles, as demonstrated in both observational and interventional studies.

In a 2016 study involving a cohort study in Poland, researchers identified an inverse relationship between VD levels and key lipid markers, including total cholesterol (TC), LDL-C, and triglycerides (TGs) [119]. A larger study of more than 20,000 participants further confirmed a significant correlation between VD deficiency and an atherogenic lipid profile [120]. Recent meta-analyses have expanded on these findings. A 2015 meta-analysis of eight randomized controlled trials (RCTs) demonstrated that VD supplementation reduced TG levels and increased high-density lipoprotein cholesterol (HDL-C), though it was also associated with elevated levels of LDL-C, with observed variability in outcomes and dosage [121].

A more extensive meta-analysis that included 39 RCTs found a significant inverse relationship between VD supplementation and TG, TC, and LDL-C levels, along with an increase in HDL-C [122]. Similarly, another meta-analysis of seven RCTs examining co-supplementation of VD and calcium in overweight and obese patients showed that less than eight weeks of supplementation led to notable reductions in TG, TC, and LDL-C, as well as an increase in HDL-C levels [123].

One of the first studies on this topic, conducted in 1992 in Belgium, involved 358 subjects and measured various biomarkers, including total serum calcium, 25-hydroxyvitamin D (25OHD), TC, HDL-C, apolipoproteins A-I (apoA-I) and B (apoB), and total protein [124]. This study identified a substantial positive connection between serum levels of 25OHD and HDL-C as well as apoA-I, indicating that higher VD levels were associated with favorable lipid profiles, particularly higher HDL-C, which is known for its protective cardiovascular effects. Subsequent cross-sectional studies with large populations have reinforced these findings, reporting significant inverse correlations between 25OHD levels and TGs and LDL-C, and a favorable connection with HDL-C as Jiang in 2018 [125]. A 2007 study by Botella-Carretero in 73 morbidly obese patients found that VD deficiency was prevalent in 50.7% of the cohort, with those deficient in VD showing lower HDL-C and higher TGs [126].

The influence of VD on lipid profiles has been examined in populations at elevated cardiovascular risk, including individuals with type 2 diabetes mellitus (T2DM). A meta-analysis conducted by Jafari et al., encompassing 17 studies that compared VD administration with placebo in individuals with T2DM, revealed that VD markedly enhanced serum levels of TC, TGs, and LDL-C, although changes in HDL-C were not statistically significant [127]. This indicates that VD may positively influence lipid profiles in individuals with diabetes, potentially through its role in regulating insulin resistance.

However, several observational and interventional studies have yielded ambiguous results. Due to inconsistent findings on the effect of VD on lipid profiles, a recent study conducted in 2023 aimed to explore this issue in more detail [128]. The results of this umbrella meta-analysis support the hypothesis that VD supplementation can positively impact lipid profiles and may serve as an effective dietary intervention to manage dyslipidemia. According to the study, VD significantly reduced TG levels but did not have a significant effect on LDL cholesterol levels. The impact of VD on HDL cholesterol and TC levels showed conflicting results. Subgroup analyses indicated that doses greater than 4000 IU per day and intervention durations shorter than 12 weeks could be critical factors influencing these outcomes.

Table 2 below provides a summary of observational and interventional studies investigating the impact of VD supplementation on lipid profiles in individuals with T2DM, prediabetes, and metabolic syndrome, the latter predisposing individuals to the development of diabetes.

## 5. Vitamin D Supplementation and T2DM Complications: Standalone Benefits Versus Combined Therapeutic Strategies

### 5.1. Macrovascular Complications

The influence of vitamin D on the risk of cardiovascular disease and diabetes-related problems continues to be a subject of significant contention. A recent randomized controlled trial involving over 25 thousand individuals demonstrated that vitamin D treatment over a 5-year duration did not have a statistically significant protective benefit against cardiovascular events [156]. However, the validity of these findings may be compromised by selection bias, as the study cohort mainly consisted primarily of individuals without VD deficiency [157]. On the contrary, other research has produced divergent results. Meta-analyses suggest that hypovitaminosis D may actually mitigate cardiovascular risk [122,158]. Furthermore, clinical investigations have correlated VD deficiency with indicators of cardiovascular impairment, such as dysfunction of endothelial cells, assessed through flow-mediated dilation of the brachial artery, and heightened carotid intima-media thickness [159,160]. Mechanistic studies further underscore the essential role of VD in vascular protection and regeneration, demonstrating that normalization of VD levels can lead to marked improvements in markers of vascular function [161,162,163,164,165]. Additionally, VD has been documented to have protective benefits against cerebrovascular issues [166].

In 2016, Wu et al. published the first randomized study that examined the effects of VD supplementation in patients with coronary artery disease. The study found that patients who received VD supplementation experienced greater reductions in the activity of the high-sensitivity C-reactive protein and renin–angiotensin system, together with improvements in the SYNTAX score (SYNergy between the percutaneous coronary intervention with TAXus and cardiac surgery) [167]. The Randomized Evaluation of Calcium Or VD (RECORD) trial, involving 5262 elderly participants assigned to receive cholecalciferol (800 IU daily), calcium (1000 mg daily), a combination of both, or a placebo, revealed a significant decrease in heart failure (HF) events associated with vitamin D supplementation relative to no supplementation. However, the trial did not identify substantial differences in the occurrence of myocardial infarction (MI) or stroke [168]. Similarly, The VitamIN D treating individuals who have Chronic heArT failurE (VINDICATE) study—a randomized, double-blind, controlled trial involving elderly individuals with HF and VD deficiency—demonstrated that daily supplementation with 100 μg (4000 IU) of cholecalciferol resulted in a significant enhancement of left ventricular structure and function [169]. Notwithstanding these favorable results, when evaluating hard outcomes like mortality, VD intake in HF-deficient individuals did not demonstrate significant differences compared to placebo [170].

Three major randomized controlled trials have examined the impact of vitamin D supplemental intake on cardiovascular ailments outcomes as the primary outcome: the Vitamin D Assessment (ViDA) trial, the Vitamin D and Omega-3 Trial (VITAL), and the Vitamin D3–Omega-3–Home Exercise–Healthy Aging and Longevity (DO-HEALTH) trial [156,171].

The ViDA study involved 5108 participants between the ages of 50 and 84 years who were randomized to receive either a per month bolus dosage of 100,000 IU of cholecalciferol or a placebo. During a median follow-up duration of 3.3 years, the primary endpoint, defined as the composite incidence or mortality from cardiovascular disease, was observed in 11.8% of the participants in the intervention group and 11.5% of the group receiving the placebo, demonstrating no meaningful differences. Similar findings were observed regardless of the baseline VD status and across secondary outcomes. The VITAL study involved 25,871 participants, with men 50 years and older and women 55 years and older. Individuals were randomized to receive a daily dosage of 2000 IU of cholecalciferol and 1 g of omega-3 fatty acids, with placebo controls for both supplements. The median duration of the follow-up period was 5.3 years. The primary outcomes included invasive cancer of any type and serious adverse cardiovascular incidents, which were defined as an aggregate of myocardial infarction (MI), stroke, or death from cardiovascular causes. The experiment did not demonstrate a substantial decrease in the occurrence of serious adverse cardiovascular incidents or invasive malignancy. The DO-HEALTH study enlisted 2157 adults 70 years and older, using a factorial 2 × 2 × 2 design to assess the effects of cholecalciferol supplementation, omega-3 enhancement, and a resistance exercise regimen. Participants received 2000 IU of cholecalciferol daily. Over a median follow-up period of 2.99 years, the primary endpoints, systolic and diastolic arterial pressure as they relate to cardiovascular diseases, did not show statistically substantial disparities between the cholecalciferol supplementation group and the placebo group. Collectively, these three large, randomized trials did not show any clinical benefit of VD supplementation on cardiovascular results, notwithstanding changes in supplement dose and duration of subsequent follow-up visits. These findings are further corroborated by meta-analyses, which indicate that while VD supplementation is safe, it has no significant impact on cardiovascular health [172,173].

The effects of VD supplementation on hypertension remain vague. Although certain studies indicate that high-dose VD supplementation can lead to short-term improvements in blood pressure, a meta-analysis of sixteen clinical studies revealed no significant effect of VD on arterial blood pressure, except in patients with existing cardiometabolic disorders [174]. For younger individuals with VD deficiency, the impact of supplementation on arterial pressure is negligible [175]. Regarding the lipid profile, a meta-analysis of thirty-eight randomized clinical studies revealed a modest but statistically substantial reduction in total cholesterol, LDL, and triglycerides, with a rise in HDL levels in participants receiving VD supplementation compared to placebo [122]. A more recent meta-analysis conducted by Dibaba, which included 41 randomized controlled trials involving 3434 individuals, confirmed similar effects on TC, LDL, and TGs. However, HDL serum levels were not significantly altered with VD supplementation in this analysis [176]. Additionally, evidence suggests a beneficial interaction between statins and VD concentrations. The implementation of high-concentration statins for individuals experiencing percutaneous coronary intervention (PCI) has been shown to independently increase VD levels, regardless of supplementation, and to reduce platelet reactivity, providing additional cardiovascular benefit [177].

### 5.2. Microvascular Complications

#### 5.2.1. Diabetic Nephropathy

Diabetic nephropathy is one of the most prevalent causes of chronic kidney disease, with microalbuminuria and proteinuria serving as critical biomarkers of this condition [178,179]. A meta-analysis of nine RCTs revealed a positive, although not statistically significant, trend towards a reduction in albuminuria facilitated by VD, indicating a potential role for VD in decelerating the progression of diabetic nephropathy [180]. Moreover, a significant disparity in 25(OH)D levels has been consistently observed in patients suffering from painful diabetic peripheral neuropathy [181]. Intervention with VD in these patients has been correlated with a marked alleviation of neuropathic symptoms [182]. The meta-analysis by Derakhshanian et al. explored the association between VD status and diabetic nephropathy, as well as the impact of VD repletion [183]. This systematic review identified an inverse relationship between serum 25-hydroxyvitamin D (25OHD) levels and the risk of diabetic nephropathy. Despite this correlation, trials investigating VD supplementation did not reveal a significant effect on urine albumin-to-creatinine ratio (UACR). These findings suggest that while VD deficiency is associated with an elevated risk of nephropathy in individuals with T2DM, current clinical trials do not support a direct causal relationship.

The meta-analysis by Chokhandre et al. focused on the supplementation of VD and its analogs in the context of chronic kidney disease (CKD), a condition intrinsically related to diabetic nephropathy [184]. Given the importance of oxidative stress as well as inflammation in the pathophysiology of diabetic nephropathy, which is modulated by VD receptor activity, the meta-analysis assessed renal outcomes such as UACR, albuminuria, and estimated glomerular filtration rate (eGFR). In particular, VD analogs such as cholecalciferol, calcitriol, and paricalcitol demonstrated a significant improvement in renal function in two RCTs, possibly due to their ability to bypass the renal-dependent 1-α-hydroxylation process. However, more extensive RCTs are required to establish their efficacy and safety profile.

Li’s review further underscored that diabetic nephropathy is a leading cause of kidney failure in diabetic patients, emphasizing the pivotal role of the renin–angiotensin–aldosterone system (RAAS) in the progression of kidney damage [185]. Although RAAS antagonists are a cornerstone in the treatment of diabetic nephropathy, their effectiveness is often hampered by a compensatory increase in renin levels. VD has been shown to exert a renoprotective effect by down-regulating RAAS by suppressing renin expression. In fact, mice lacking the VD receptor exhibit exacerbated diabetic nephropathy, indicating that impaired RAAS regulation is a contributing factor. When combined with RAAS inhibitors, VD analogs significantly mitigate renal injury by preventing a compensatory increase in renin.

In conclusion, low VD status is associated with decreased renal function in patients with T2DM and CKD. Although VD supplementation with VD alone has not shown a definitive causal impact on improving renal outcomes, the use of VD analogs, particularly when combined with RAAS inhibitors, shows promise in improving renal function in this population.

#### 5.2.2. Diabetic Peripheral and Automatic Neuropathy

Diabetic peripheral neuropathy (DPN) affects up to 50% of people with diabetes, and about 30% show symptoms [186,187]. A UK study of 15,692 patients revealed that 34% of South Asians and 21% of Europeans experienced painful DPN, indicating ethnic disparities [188]. Emerging evidence suggests that VD may have analgesic and neuroprotective effects, with deficiency associated with various pain syndromes and peripheral neuropathy in conditions such as primary Sjögren’s syndrome [189]. A meta-analysis including 1484 T2DM patients found that VD deficiency significantly increases the risk of DPN [190]. An open-label trial showed that 2059 IU of oral cholecalciferol daily for three months reduced pain by 50%, surpassing the typical placebo effect of 30% [191]. However, a study of the QR-333 VD analog showed mixed results due to the inclusion of quercetin, which complicates interpretations [192].

A case-controlled research study involving 150 individuals with DPN and 600 involved in the control group reported a complex and non-linear relationship between serum 25-dihydroxyvitamin D (25[OH]D) and symptomatic DPN. Reduced levels of 25(OH)D (<20 ng/mL) were correlated with a higher risk of symptomatic DPN, while very high levels (>40 ng/mL) were correlated with an even greater risk, highlighting the need for careful monitoring [193]. In Egypt, a study of 40 patients with DPN found a significant association between VD insufficiency and the severity of DPN, with lower levels of 25(OH)D in patients with painless neuropathy [194]. A French study confirmed that patients with DPN are typically older, have a longer duration of diabetes, and have lower VD levels [195]. Another cross-sectional study of 136 participants found lower 25(OH)D levels in patients with PND and identified VD deficiency (<20 ng/mL) as a significant PND predictor, along with the duration of diabetes [196].

Lee et al. observed a notable association between VD deficiency and diabetic peripheral neuropathy (DPN) in a cohort study of 51 individuals with T2DM, all of whom had low serum levels of 25-dihydroxyvitamin D (25[OH]D) and painful neuropathy [191]. Following a three-month regimen of VD supplementation, a 50% reduction in neuropathic pain scores was recorded. In a case report by Bell et al., a 38-year-old man with a 27-year history of type 1 diabetes mellitus (T1DM) and a decade-long history of neuropathic symptoms experienced severe pain that required potent analgesics [197]. With an initial 25(OH)D level of 16.5 ng/dL, the patient received VD supplementation. Correction of the deficiency led to rapid relief of neuropathy symptoms and a substantial reduction in analgesic requirements. A comparative study involving 87 T2DM patients with DPN and 123 without revealed that the former group had significantly lower serum 25(OH)D levels [198]. Supplementation with VD in these patients culminated in a significant decrease in both symptoms and signs of DPN.

Recent research focused on evaluating the efficacy and tolerability of VD supplements for painful DPN including 66 T2DM patients with painful neuropathy. Participants received weekly doses of 50,000 IU of vitamin D3 over a period of 12 weeks [199]. This treatment led to notable increased serum levels of 25(OH)D and a substantial decrease in DPN symptoms and signs. In a more recent study, the impact of VD supplementation on microcirculation, DPN symptoms, and inflammatory markers in T2DM patients was assessed [200]. High-dose VD therapy was found to reduce serum concentrations of the pro-inflammatory cytokine interleukin-6 and increase serum concentrations of the anti-inflammatory cytokine interleukin-10. These changes were associated with improvements in the severity of DPN and skin microcirculation.

Data on the link between cardiovascular autonomic neuropathy (CAN) and VD deficiency are limited. Some cross-sectional studies suggest associations between 25(OH)D levels and the presence and severity of CAN in patients with diabetes [201,202]. VD receptors are located in smooth muscle cells in the vascular system, the endothelial tissue, and cardiomyocytes, with insufficiency associated with cardiovascular disorders, tumors, autoimmune disorders, overall mortality, and possibly diabetes and neurodegenerative diseases [203,204,205]. CAN itself may increase the risk of cardiovascular mortality [206]. Supplementation with VD supplementation has improved CAN measures in people without diabetes [207].

Low heart rate variability, which can signal cardiovascular disease, was studied in relation to VD status in 163 patients with T2DM using five cardiovascular reflex tests and heart rate variability metrics [208]. Participants were classified according to 25(OH)D levels: sufficient (≥20 ng/mL), insufficient (10–20 ng/mL), or deficient (<10 ng/mL). VD deficiency showed a significant correlation with heart rate variability, though its association with CAN was borderline significant, warranting further investigation.

#### 5.2.3. Diabetic Retinopathy

VD levels are related to the optic chiasm volume and several studies indicate a connection between VD deficiency and age-related macular degeneration (AMD) [209,210,211]. In a Turkish study, an inverse correlation was found between worsening diabetic retinopathy and lower concentrations of 1,25-dihydroxyvitamin D_3_ (active VD) [212]. In this study, the lowest levels of VD were observed in cases of proliferative DR, while the highest levels were observed in patients with diabetes but without DR. A study of 50 patients with diabetes and early-stage retinopathy found that VD deficiency was associated with thinning of the retinal nerve fiber layer (RNFL) [213]. Similarly, in a Korean study of 18,363 NHANES participants, 25(OH)D concentrations were associated with diabetic retinopathy, with corroborating findings in the Chinese population with diabetes [214,215]. However, a third NHANES study and the EURODIAB prospective complications study found no significant relationship between 25(OH)D and the severity of the retinopathy, although EURODIAB showed a positive correlation with microalbuminuria [216,217]. Recent research by Alam et al. also found no association between 25(OH)D levels and diabetic retinopathy or maculopathy, possibly due to high rates of deficiency among their subjects (91% with 25(OH)D <30 ng/mL) [218]. Zoppini et al. identified a significant association between serum 25-hydroxyvitamin D [25(OH)D] levels and the prevalence of diabetic retinopathy (DR) in a cohort of 715 patients with type 2 diabetes [219]. Similarly, Alcubierre et al. found that patients with advanced DR exhibited lower serum 25(OH)D concentrations compared to those without DR [220]. Zhao et al. reinforced this association in a meta-analysis, demonstrating that patients with type 2 diabetes and VD deficiency (defined as serum 25(OH)D levels below 20 ng/mL) had a significantly elevated risk of developing DR [221]. These findings underscore a link between VD deficiency and an increased risk of DR in individuals with T2DM.

It is important to note that current evidence suggests that optimal magnesium status plays a significant role in optimizing VD status. Magnesium is a cofactor for enzymes involved in the metabolism of VD, including its conversion into its active form, 1,25-dihydroxyvitamin D. Magnesium facilitates the conversion of vitamin D into its bioactive forms, thereby enhancing its effectiveness in the body. Specifically, some studies suggest that magnesium supplementation increases 25(OH)D3 concentrations when baseline VD levels are close to 30 ng/mL but decreases them when baseline levels range from 30 to 50 ng/mL [222]. An RCT conducted in 2022 found that a combination of magnesium and vitamin D supplementation may be more effective in raising serum 25-hydroxyvitamin D levels compared to VD alone, particularly in overweight or obese individuals [223]. Further research is required to explore the co-administration of VD with other minerals, such as magnesium and calcium, to better understand their combined effects.

## 6. Conclusions

VD influences the pathophysiology of diabetes by enhancing insulin synthesis and secretion, improving insulin sensitivity, and reducing oxidative stress, inflammation, and DNA methylation. The impact of VD supplementation on glucose metabolism and the incidence of T2DM has yielded mixed results across various clinical trials. Furthermore, VD levels are correlated with unfavorable serum lipid profiles, while adequate VD levels are associated with healthier lipid profiles. VD supplementation has demonstrated a beneficial effect on the progression of diabetes-related complications.

However, the evidence is particularly convincing regarding the benefits of supplementation in the prevention of diabetes, and this is reflected in the latest guidelines of the Endocrine Society which recommend empiric vitamin D supplementation in high-risk adults to reduce the risk of progression to diabetes. In all other cases, research is ongoing, and more studies are needed before the broad implementation of vitamin D supplementation for the prevention and management of diabetic complications [224].

Finally, the considerable variability observed among studies and clinical trials—including differences in patient demographics, baseline vitamin D status, supplementation dosages, and the selection of primary endpoints—complicates the formulation of definitive conclusions and robust clinical recommendations. While preclinical studies and observational data often highlight a protective role for vitamin D in the context of diabetes, the inconsistencies present in both randomized and nonrandomized interventional studies underscore the necessity for further exploration of the relationship between vitamin D and glucose metabolism. This research should include genetic studies to provide more nuanced insights into individual responses to vitamin D supplementation. Such investigations will be essential for the design and execution of more targeted and effective randomized clinical trials tailored to the specific characteristics of relevant patient populations.

## Figures and Tables

**Table 2:** Observational and interventional studies investigating the effects of vitamin D supplementation on serum lipids.

Observational Studies			
Study	Study/Country	Population	Results/Outcomes
Karhapää P. et al. (2010) [129]	Observational/Finland	n = 909, T2DM	Reduced levels of 1,25-dihydroxyvitamin D were correlated with diminished HDL-C levels, whereas low levels of VD were connected with elevated TC, LDL-C, and TGs.
Huang, Y. et al. (2013) [130]	Cross-sectional/China	n = 2652, T2DM	VD serum levels were positively correlated with lipoprotein lipase.
Saedisomeolia, A. et al. (2014) [131]	Cross-sectional/Iran	n = 108, T2DM	Individuals with VD deficiency showed elevated serum concentrations of TC, TGs, and LDL-C, along with reduced concentrations of HDL-C, in comparison to those with adequate VD levels. However, a statistically significant correlation was observed only for TGs.
Wang L. et al. (2020) [132]	Case–Control/China	n = 2659, T2DM and IFG group	Maintaining sufficient vitamin D levels may reduce the incidence of T2DM by enhancing the profile of lipids.
Saheb Sharif-Askari F S et al. (2020) [133]	Cross-sectional/Arab Emirates	n = 2489, insulin resistance and insulin-sensitive group	A deficiency in vitamin D was connected to decreased levels of HDL-C.
Gong T. et al. (2022) [134]	Observational/China	n = 306, T2DM	In overweight or obese individuals with T2DM, serum VD levels showed an independent, negative correlation with TGs.
Raheem M. et al. (2022) [135]	Observational/Iraq	n = 90, T2DM and healthy	When serum VD levels were notably low, TC and TGs were notably higher in individuals with T2DM in comparison with controls.
Atia T. (2023) [136]	Cross-sectional/ Saudi Arabia	n = 145, non-diabetes n = 104, prediabetes	VD deficiency was more common in individuals with prediabetes and was linked to elevated TG levels and HDL levels, while TC and LDL levels remained unchanged.
Pathania M. et al. (2023) [137]	Single-center Cross-sectional/ India	n = 235, Metabolic Syndrome	Low levels of VD in the serum display a minimal association with TC, TGs, and LDL-C.
**Interventional Studies**			
**Study**	**Study/Country**	**Population**	**Results/Outcomes**
von Hurst P. et al. (2010) [138]	Randomized, placebo-controlled, double-blind trial/ New Zealand	n = 81, insulin resistance	VD exerted no influence on serum lipid concentrations.
Shab-Bidar S. et al. (2011) [87]	Randomized controlled clinical trial/Iran	n = 100, T2DM	VD reduced TGs, TC, and LDL-C while increasing HDL-C.
Nikooyeh B. et al. (2011) [139]	Interventional study/Iran	n = 90, T2DM	VD did not influence serum lipid concentrations.
Al-Daghri NM et al. (2012) [140]	Multi-center, interventional study/Saudi Arabia	n = 92, T2DM	VD supplementation lowered TC and LDL-C.
Wongwiwatthananukit S et al. (2013) [141]	Prospective, randomized, double-blind, double-dummy, parallel trial/ USA	n = 90, Metabolic Syndrome	VD showed no effect on concentrations of serum lipids.
Breslavsky A. et al. (2013) [91]	Randomized, double-blind, placebo-controlled study/ Israel	n = 47, T2DM	VD did not have any impact on concentrations of serum lipids.
Ryu O. et al. (2014) [142]	Prospective, randomized, double-blinded, placebo-controlled trial/ Korea	n = 62, T2DM	VD had no impact on concentrations of serum lipids.
Yousefi Rad E. et al. (2014) [103]	Randomized controlled trial study/ Iran	n = 58, T2DM	The levels of HDL-C rose substantially among both groups.
Kim HJ. et al. (2014) [105]	Interventional study/ Korea	n = 52, T2DM	The combination of VD and fitness regimen lowered TC, TGs, and LDL-C while increasing HDL-C.
Eftekhari MH et al. (2014) [143]	Double-blind randomized placebo-controlled/ Iran	n = 70, T2DM	A decline in TC, TGs, and LDL-C was noted in each of the groups; however, HDL-C diminished solely in the placebo group.
Al-Zahrani MK et al. (2014) [94]	Randomized placebo-controlled/ Saudi Arabia	n = 200, T2DM	VD did not alter serum lipid levels.
Dutta D. et al. (2014) [144]	Interventional study/ India	n = 121, prediabetes and diabetes	Vitamin D supplementation in individuals with prediabetes and diabetes correlates with enhancements in dyslipidemia.
Sadiya A. et al. (2015) [95]	Randomized double-blind clinical trial/ UAE	n = 87, T2DM and obesity	VD showed no effect on serum lipid levels.
Muñoz-Aguirre P. et al. (2015)	Randomized, double-blind, placebo-controlled/ Mexico	n = 104, T2DM	VD lowered TG levels.
Jafari T. et al. (2015) [127]	Double-blind randomized placebo-controlled trial/ Iran	n = 59, T2DM	VD did not have any impact on concentrations of serum lipids.
Ramiro-Lozano JM et al. (2015) [145]	Interventional study/ Spain	n = 28, T2DM	VD reduced TC and LDL-C.
Yin X et al. (2016) [146]	Randomized double-blind placebo-controlled/ China	n = 126, Metabolic Syndrome	VD did not affect concentrations of serum lipids.
Salekzamani S et al. (2016) [147]	Randomized, controlled, double-blind study/ Iran	n = 80, Metabolic Syndrome	VD supplementation lowered TG serum levels and the TG/HDL ratio.
Jorde R. et al. (2016) [148]	Randomized controlled Trial/ Norway	n = 511, prediabetes	In the VD group, a decrease in LDL-C occurred solely after one year.
Makariou S et al. (2017) [149]	Prospective, randomized, open-label, blinded endpoint/ Greece	n = 50, Metabolic Syndrome	VD had no impact on concentrations of serum lipids.
Liyanage GC. et al. (2017) [150]	Randomized double-blind clinical trial/ Sri Lanka	n = 85, T2DM	VD lowered TC and LDL-C while increasing HDL-C.
Riek AE et al. (2018) [151]	Interventional study/ USA	n = 26, T2DM	VD did not influence plasma lipid levels, but it diminished total monocyte cholesterol concentrations by inhibiting the uptake of oxidized LDL cholesterol.
Farag A et al. (2019) [152]	Randomized controlled trial/ Iraq	n = 70, Metabolic Syndrome	Only the combination of VD and physical activity resulted in a decrease in TC, LDL-C, and HDL-C.
Angellotti E. et al. (2019) [153]	Double-blind, randomized, placebo-controlled clinical trial/ USA	n = 127, T2DM	VD did not affect concentrations of serum lipids.
Bhatt SP et al. (2020) [90]	Open-label randomized placebo-controlled trial/ India	n = 121, prediabetes	VD did not influence concentrations of serum lipids.
Rajabi-Naeeni M. et al. (2020) [154]	Factorial, triple-blind clinical trial/ Iran	n = 168, prediabetes	In the group receiving both VD and omega-3, TC, TGs, and LDL-C levels decreased, while HDL-C levels increased. In the group taking only VD, TC, and LDL-C levels were reduced.
Misra P. et al. (2021) [155]	Open-label randomized placebo-controlled trial/ India	n = 132, prediabetes	VD did not influence concentrations of serum lipids.

TC, total cholesterol; HDL-C, high-density lipoprotein cholesterol; TGs, triglycerides; LDL-C, low-density lipoprotein cholesterol; T2DM, type 2 diabetes mellitus.
